# Effective Teaching and Examination Strategies for Undergraduate Learning During COVID-19 School Restrictions

**DOI:** 10.1177/0047239520934017

**Published:** 2020-09

**Authors:** Marcus L. George

**Affiliations:** 1 Department of Electrical and Computer Engineering, University of the West Indies, Trinidad and Tobago

**Keywords:** novel coronavirus, COVID-19, teaching during COVID-19, teaching during pandemics, teaching undergraduates, undergraduate education, online education

## Abstract

On Friday, March 13, 2020, all school teaching in the Republic of Trinidad and Tobago, West Indies was suspended until further notice because of the novel coronavirus COVID-19 pandemic. This immediately jeopardized the completion of course content at the University of the West Indies, St. Augustine campus. This article presents effective teaching and examination strategies that can be utilized in teaching undergraduates during COVID-19 school restrictions. The introductory digital electronics course of the Department of Electrical and Computer Engineering at the University of the West Indies will be utilized to demonstrate the merits of these strategies. The research will focus on demonstrating that the teaching methodologies utilized avoided the student performance from degrading below what has been experienced in the past 5 academic years. Student feedback on the methodology utilized is also incorporated in this article to highlight key benefits gained by students.

Tertiary-level education serves to provide both strong theoretical foundation as well as the capability to solve practical issues faced in the industry. Every academic year introductory courses are presented to Level 1 undergraduates at the University of the West Indies (UWI), St. Augustine campus. One such course is the introductory digital electronics course presented to students of the Department of Electrical and Computer Engineering. All students begin this course without previous knowledge and practical capabilities of digital logic design.

Traditionally, this introductory digital electronics course was delivered in the classroom via contact teaching methods; all labs, exams, and quizzes were administered in a classroom setting. In 2020, during the COVID-19, this was the methodology of teaching up until the Thursday, March 12, 2020 after which significant restrictions were placed on school teaching, and in-class delivery of course content was suspended indefinitely. Before a solution to this issue was found, it was crucial to review literature of existing teaching approaches that could have some merit in the way forward.

Nickels (2000) presented a strategy for the teaching digital electronics to undergraduates with the use of computer-aided design (CAD) tools and hardware description languages. A hierarchical strategy was utilized, and this involved having students beginning with the study of simple digital electronic circuits after which they progressed to problems involving more complex digital electronic. Nickels (2000), however, did not conclude whether the methodology implored led to any benefit in the area of student learning or performance. The merits of this teaching methodology are that it provides a step-by-step graduation of students from less difficult digital electronics problems to more difficult ones. Like digital electronics, other disciplines can benefit from the use of this teaching methodology because of the step-by-step progression of students from less difficult problems in the discipline to more difficult ones. One consideration not discussed by Nickels (2000) is that of student support, and it is expected that no matter the discipline, student support will be an invaluable resource as the difficulty of problems they attempt increases.

Weng et al. (2009) conducted a study to determine if the use of programmable logic tool kits could assist computer science students in the learning of digital electronics. Students were introduced to the use of programmable logic boards via laboratory exercises while course demonstrators monitored students’ reactions to the material administered. A comprehensive survey that captured opinions of students for the use of programmable logic boards in their learning of digital electronics was conducted by Weng et al. (2009). According to Weng et al. (2009), there was no disagreement among students that the use of programmable logic boards assisted them in learning digital electronics. Eighty-six percent of the students found the use of programmable logic board was a pleasant experience. Weng et al. (2009) also indicated that there was an improvement in student performance when programmable logic boards were used when compared with previous years when programmable logic boards were not used. This methodology provides a practical approach to teaching, and it presents opportunities for other disciplines. Programmable logic tool kits were utilized for digital electronics. For other disciplines such as mathematics, physics, biology, or even finance, tool kits appropriate for practical teaching of those subject areas can be incorporated into the curriculum to enhance student learning. For online learning, students must have access to these resources at home.

Zhao and Okamoto (2009) presented an adaptive framework aimed at engaging students in collaborative discussion of course material in ubiquitous environments. The methodology promoted student use of mobile technology to conduct email-based discussion of course material at any time during the teaching period. Zhao and Okamoto (2009) claimed that the proposed adaptive framework improved the learning experience of the students and increased student performance in the area of study. This methodology presents opportunities for lecturing of most disciplines, even during pandemics. Use of mobile technology by college students is more popular today than in the past; hence, there is merit in the use of ubiquitous environments to support teaching.

Joseph et al. (2013) presented the utilization of case method and role-play in teaching the topic of finite state machines (FSMs) to undergraduates. Joseph et al. (2013) indicated that the case method is normally used as a very important pedagogical tool in academia, and its use is intended for the enhancement and development of the general conclusions of the research being done. Joseph et al. (2013) indicated that role-play is less technologically elaborate and is utilized for the learning interpersonal skills. Joseph et al. (2013) claimed students displayed greater interest in the use of the case method and role-play techniques for learning of course topics and that there was an increase in the level of collaboration and active participation by students in the learning process. Finally, Joseph et al. (2013) claimed that student performance was better when case method and role-play techniques were utilized compared with when they were not utilized. This methodology can benefit the teaching of most disciplines simply because it increases interaction between students in the learning process without a demand on the use of discipline-specific resources. This is especially beneficial to courses containing group projects.

Prasad et al. (2014) conducted a study of the impact of software simulators in the teaching of digital logic design. The tools Logic Gate Simulator, Digisim, TinyCAD, and Logisim were utilized in this study. Prasad et al. (2014) claimed that the use of logic simulators such as Logic Gate Simulator, Digisim, TinyCAD, and Logisim resulted in the number of students scoring above 45% in the course increasing from 82% in the previous year to 96% in the present year. This methodology provides opportunities for expanding on the teaching of topics in any discipline such as engineering, mathematics, finance, or even geography, as long as the appropriate simulators are made available. The use of simulators can also support self-study by students at home as long as the software is stand-alone.

Montãnanaa et al. (2015) discussed the use of participative learning in teaching of very high speed integrated circuit hardware description language (VHDL). This methodology gave the students the freedom to think and explore and hence allowing them the opportunity to discover better methods of learning the topic. At the end of the study, students indicated that the appropriate planning, distribution, and clear definition of the specifications of project tasks can eliminate the possibility of conflicts in the design and implementation stages of projects. Montãnanaa et al. (2015) also indicated that students appreciated the need for teamwork in managing large projects when this methodology was utilized. The strength of the methodology presented by Montãnanaa et al. (2015) is the freedom to participate in learning without restrictions. Students are introduced to the topics, given objectives, and allowed the freedom to explore the most appropriate methods of learning the topics. This can benefit the teaching of most disciplines especially because the method utilizes no discipline-specific resources.

Roy et al. (2015) presented the use of web-based virtual laboratory called COLDVL in the teaching of the topic of computer organization and logic design. COLDVL contains a hierarchical module-level logic tool that contains a logic simulator component and also a large number of technical features. The system also consists a graphical user interface that can be used in the construction and simulation of logic circuits. Roy et al. (2015) was unable to indicate whether the use of COLDVL enhanced student learning of the topic. Like Prasad et al. (2014), this methodology provides opportunities for expanding on the teaching of topics in any discipline as long as the appropriate software is made available to students. This methodology will promote self-study by students, and consultation opportunities must be available to guarantee student progress.

George (2018) presented the exploration of a classroom-based methodology for teaching of digital logic to engineering undergraduates at the UWI, St. Augustine campus. In the classroom-based methodology, all lab work was conducted inside the classroom rather than the laboratory. To assess the merits of students under the new approach, the performance of students when this classroom-based teaching methodology was utilized was compared with that of the previous lab-based teaching methodology. George (2018) indicated that students’ performance was better in all quizzes and design project when this classroom-based teaching methodology was utilized when compared with the previous teaching methodology. Students also endorsed the use of this classroom-based methodology in teaching digital logic design. The merit of this teaching methodology is that all laboratory work was conducted in a classroom setting and not a laboratory, hence allowing the lecturer the opportunity to incorporate practical work while presenting course content. If a course is to be conducted online, this teaching methodology can be utilized as long as a video conference platform is utilized and students themselves have access to development resources such as the field programmable gate array (FPGA) tool kits.The methodology of George (2018) however only benefits courses with practical components and as such other courses such as English Language and History which normally would not require the use of a laboratory may not receive any additional benefits from this methodology.

George (2020) presented a study on the effect of using three consultation types—office, email, tutorial—in the teaching of FSMs to Electrical and Computer Engineering undergraduates at the UWI, St. Augustine campus. George (2020) made the conclusion that students who participated in consultation activities offered by the course lecturer performed better in FSM-related quizzes and projects than students who did not participate in such in consultation activities. George (2020) also concluded failures in the quizzes occurred only in the population of students who attended class lectures alone but never participated in consultation exercises. This methodology provides many opportunities for lecturers of any discipline to provide different levels of support to meet the varying needs of students. In a pandemic, however, the tutorial-based and office-based consultations will only be possible via video conferencing. The email-based consultations will not be affected regardless of the existence of pandemic and regardless of the discipline being taught.

With the consideration of the fact that COVID-19 school restrictions were in place in Trinidad and Tobago for the remainder of second semester year 2019/2020, it was important to review the traditional approach to delivering the introductory digital electronics course and determine what was possible and not possible under the circumstances. Then, the merits and drawbacks of the literature reviewed earlier can be used as a guide to developing a modified strategy to completing the delivery and assessment of the course despite COVID-19 teaching restrictions. Student performance using these strategies will be evaluated to determine if they were effective in avoiding degradation below what has been experienced in the past five academic years, despite the COVID-19 school restrictions. It is expected that the study will provide a basis in which other disciplines can benefit from for teaching of undergraduates during COVID-19 school restrictions.

## Summary of Traditional Teaching Methodology for Level 1 Digital Electronics Course at the UWI

The introductory digital electronics course provided students with a firm foundation in the concepts of digital logic analysis and design. The course covers topics such as number systems, Boolean algebra, minimization using Karnaugh maps, combinational logic circuits, and integrated circuit technology. Although this is an introductory course in digital logic analysis and design, the course also served to expose students to practical tools and devices used in the development of digital circuits such as the use of VHDL and Xilinx ISE. On completion of the course, students must be capable of constructing, analyzing, verifying, and troubleshooting digital circuits using appropriate techniques and test equipment.

The previous teaching methodology for the undergraduate introductory digital electronics (ECNG1014) was utilized over a 12-year period from academic year 2007/2008 to year 2019/2020. Because this was an introductory course in digital electronics, students always entered the course with little or no experience in digital logic design. The learning outcomes of the course are given in Table 1.

**Table 1.** Summary of the Learning Outcomes of the Digital Electronics Course.

LO #	Learning outcome	Cog. level
1	Demonstrate competence in the representation of information in a digital systems	C
2	Apply Boolean algebra to the design of combinational logic circuits	C, Ap
3	Apply Karnaugh maps to the design of combinational logic circuits	C, Ap
4	Describe the operation of combinational logic circuits such as comparators, encoders, decoders, multiplexers, demultiplexers, adders, and subtractors	C
5	Explain the basic operational characteristics and parameters of integrated circuits, including an understanding of CMOS and TTL technologies	C
6	Apply CMOS Conduction Complements in the construction of combinational logic circuits using MOS transistors	C, Ap
7	Demonstrate competence in the implementation and verification of digital systems using CAD tools (Xilinx ISE) along with programmable logic platforms	C, Ap

*Note*. C = comprehension; Ap = application; CAD = computer-aided design; CMOS = complementary metal-oxide-semiconductor; TTL = transistor-transistor-logic; MOS = metal-oxide-semiconductor; ISE = integrated synthesis environment.

The course was intended for delivery by 3 contact hours of lectures per week for 12 weeks (total of 36 contact hours). The course was assessed via two quizzes, one midterm exam and one final exam as shown in Table 2. The 36 hours of contact with students included 27 hours of class lectures, 5.5 hours allocated to delivering Lab #3 using of classroom-based lab delivery using the approach entailed in (George, 2018), one 90-minute slot allocated to midterm exam, and two 60-minute slots allocated to Quiz #1 and Quiz #2. Students were required to attempt Labs #1 and #2 by themselves using a detailed laboratory manual, a Nexys3 tool kit available from the laboratory facility and Digilent (2016). Students were expected to progress in these two labs without issue, but if any issues were faced, the course lecturer was available to assist using the approach of George (2020).

**Table 2.** Assessment Artefacts of the Digital Electronics Course.

Parameter	Mode	Weighting
*Lab #1—*Intro to Xilinx schematic editor	Guided self-study	0%
*Lab #2—*Combinational logic circuits	Guided self-study	0%
*Lab #3—*Introduction to VHDL	Classroom	0%
*Quiz #1—*Combinational logic circuits	Online	8%
*Quiz #2—*Introduction to VHDL	Classroom	12%
Midterm exam	Classroom	20%
Final exam	Classroom	60%

The Labs 1 to 3 did not contribute to the course mark but instead served the purpose of informing and preparing students for the quizzes that carried marks, and most important, these labs contributed to the students’ practical awareness of topics in the course.

## Description of Modified Teaching Methodology Prepared for COVID-19 School Restrictions

The class enrollment for academic year 2019/2020 was 76 students. The introductory digital electronics course presented in year 2019/2020 consisted of the same learning outcomes of previous offering in years 2015/2016 to 2018/2019 (see Table 1). Unlike previous years, the offering of 2019/2020 consisted of two different delivery phases (Table 3).

**Table 3.** Delivery Phases for the Digital Electronics Course.

Phase	Description	Period begin	Period end
1	Before COVID-19 restrictions	January 21, 2020	March 12, 2020
2	During COVID-19 restrictions	March 13, 2020	May 8, 2020

At the beginning of the semester, the course lecturer made available for purchase of a concise workbook specifically designed for the course (George, 2019). It was expected that this textbook serve as an *assistant teacher* for students of the course, allowing the opportunity to obtain detailed solutions and explanations of digital electronic problems. Of the 76 students, 67 enrolled in the course secured copies of the textbook. This will be expanded on later on in this section of the article. Also at the beginning of the semester, the traditional teaching strategy was executed including an investment in effective in-class teaching, labs conducted via the method presented in George (2018), and a variety of consultation activities as presented in George (2020).

However, on Friday, March 13, 2020, when COVID-19 teaching restrictions were imposed in Trinidad and Tobago, in-class teaching was stopped, and the modified teaching strategy was enforced. The following are the elements of the modified teaching strategy:

### a. Use of MyElearning as the Online Teaching Platform

The MyElearning online platform was utilized over the years as an avenue for students to access all course materials including lectures, lab manuals, and so forth. The advent of COVID-19 teaching restrictions made MyElearning a crucial resource for continued support of students. Even examination activities were conducted on this platform after the COVID-19 restrictions. More information on these items will be presented in the upcoming sections.

### b. Digital Logic Theory for Engineers Classic Workbook

A very useful resource in times of teaching restrictions is that of a very detailed workbook-type text. Many textbooks do not adequately meet the needs of an introductory digital electronics course. As such, the lecturer of the course has a responsibility to provide a concise text that meets the needs of his/her course. George (2019) has written a text to comprehensively cover the contents of the introductory digital electronics courses for most Electrical and Computer Engineering Undergraduate Degrees. The text presented material for all topics of the course with the aid of step-by-step solution of problems and diagrams. Practice questions along with answers to them were presented on conclusion of each chapter. As indicated previously, this textbook served as an *assistant teacher* for students. During COVID-19 teaching restrictions, the course lecturer was able to easily refer students to aspects of George (2019) for detailed explanation on problems encountered.

### c. Digital Electronics Visual Tutor

**Figure 1.**
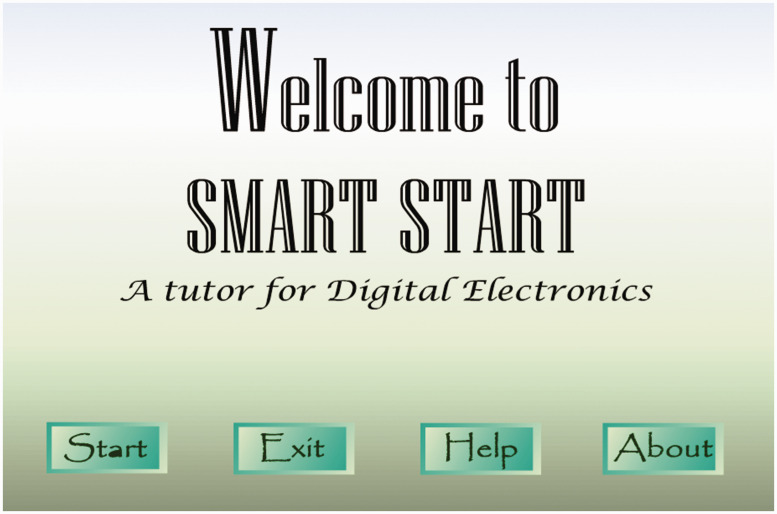
Homepage of the Digital Electronics Visual Tutor.

The digital electronics visual tutor (Figure 1) was a fantastic learning resource provided to students after the COVID-19 restrictions were placed. This visual tutor was the result of a final year project supervised by the course lecturer and was developed for this introductory digital electronics course. The course lecturer did not intend to make use of this resource for teaching the cohort of 2019/2020, neither was the resource utilized in the teaching of the last eight cohorts of Level 1 students in the department. Because of the COVID-19 restrictions, the course lecturer made this resource available for download from the MyElearning course page. This visual tutor provided an interactive learning experience of all topics of the introductory digital electronics course for all students who utilized it. This was especially useful in students learning the last two topics of the course that were not delivered before the COVID-19 restrictions: Topic #5—Introduction to VHDL and Topic #6—Integrated Circuit Technology.

### d. Port-Mapping Tool for Digital Logic Design

**Figure 2.**
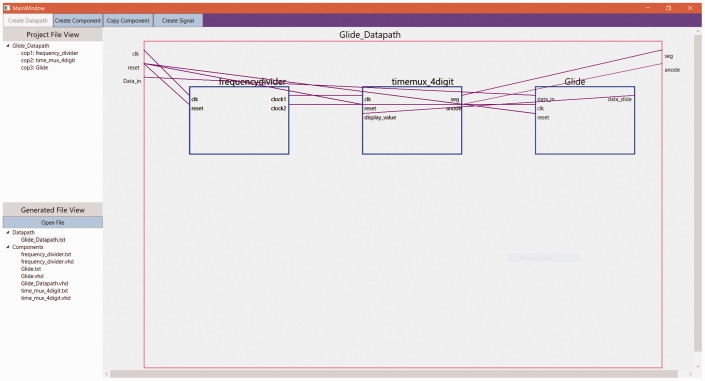
User Interface of the Port-Mapping Tool for Digital Logic Design.

Like the digital electronics visual tutor of (c), this Port-Mapping tool was a result of a final year project supervised by the course lecturer and has been identified in George and Bissoon (2019)—image seen in Figure 2. Students of the introductory digital electronics course were supposed to be introduced to VHDL, of which port mapping (component instantiation) was the most important aspect of the material to be presented. To further support student practical learning of the topic of port mapping, this Port-Mapping tool was made available for student download from the MyElearning course page. With this resource, students could have taken any datapath block diagram and use the step-by-step teaching feature to arrive at the complete VHDL code for the port mapping of any system.

### e. Selection of Online YouTube Videos

Immediately after the COVID-19 school restrictions were enforced, the majority of the population of lecturers who continued teaching moved teaching online and attempted to use the obvious online methods to deliver course material to students. Some invested time in creating online videos for upload, while some invested their time in the use of software such as Zoom and Blackboard Collaborate. Although these approaches provided a convenient alternative to the in-class lectures, they introduced several drawbacks not experienced in the in-class lecture approach as indicated in Kebritchi et al. (2017) and NBC News(2020).

Because of the variety of support resources offered by the lecturer of this introductory digital electronics course, a decision was made to locate the best available YouTube videos to support students learning of the last two topics that were not completed prior to the COVID-19 restrictions: Topic #5—Introduction to VHDL and Topic #6—Integrated Circuit Technology. The URL of these videos were posted on the MyElearning course page for students to click and view. This activity avoided the course lecturer wasting crucial time in creating videos of holding online class sessions which may be plagued with issues of online teaching indicated in Kebritchi et al. (2017) and NBC News(2020). The lecturer was hence able to maximize time invested in email-based consultations with students.

### f. Email-based Consultation

As indicated in George (2020), this consultation type involved the course lecturer presenting students with a tutorial or supplementary with topics associated with the course topics. Students were required to attempt questions on their own time in the comfort of their home, scan their attempts, and email them to the course lecturer for review and correction. The course lecturer then presented the students with a summary of corrections to their attempts via an email reply. Students who had started working on questions but unable to finish were also required to scan their attempts and email the lecturer for his review. The lecturer would then email the students on mistakes they made and the required corrections, after which they were given another opportunity to attempt the questions again and return for a second email consultation with the lecturer.

### g. Supplementary Sheets

Students were presented with supplementary worksheets at the beginning of the semester; however, the need for this was not apparent until there were no longer any in-class lectures because of COVID-19 teaching restrictions. When there were no restrictions, students could have completed the supplementary sheet for each topic, meet with course lecturer, and have office consultation as indicated in George (2020). However, because face-to-face meetings were no longer possible, students were able to use email-based consultation (George, 2020) as indicated in (f) described earlier. Students were required in this case to attempt supplementary sheets, scan their solutions, and submit via email for the lecturer’s review. The lecturer then reviewed the solutions and then set up an email-based consultation with the students to discuss any mistake made and entertain any questions.

### h. Online Mock Quizzes

**Figure 3.**
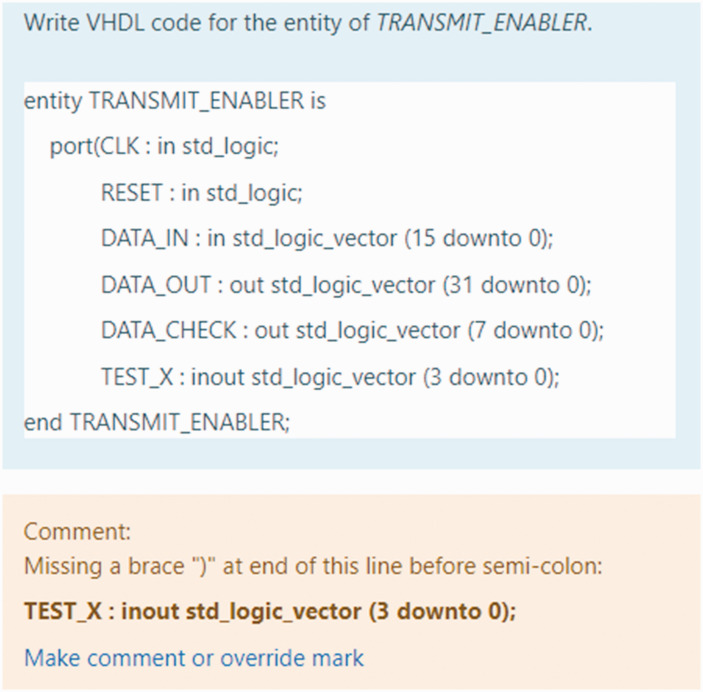
Structured MyElearning-Based Quiz Student Response and Lecturer Feedback Comments Added.

Online mock quizzes were issued to students to provide students with an opportunity to attempt structured exam-type questions for the topics of the course and to give students experience in attempting exams online in the event that an online approach was to be utilized for examination of students for the remainder of the course semester. As a matter of fact, it was the intention of the course lecturer to host all remaining exams online because it was expected that the school restrictions may have remained in place for the rest of the semester, and hence in-class exams would not have been possible. These quizzes were arranged as structured essay-type questions on the MyElearning course page, and students were required to respond to questions by placing solutions in fields provided. If students were required to provide illustrations, they were allowed to use computer programmes such as Microsoft Paint, Microsoft Visio, and then upload images to fields provided in the exam. Students were allowed multiple attempts of the quizzes, and when completed, they were manually graded. A very important part of this resource is that the quizzes allowed for the lecturer to insert comments on the student attempts so students can learn from mistakes made (see Figure 3). The provision of lecturer comments (feedback) was expected to add value to the student learning experience during the mock exam.

### i. Mock Quiz Feedback Document

To ensure that all students were allowed the opportunity to learn from each other’s mistakes and lecturer feedback, what is called a Mock Quiz Feedback Document was developed for each mock quiz provided. At least 10 students’ responses for each question administered in the quizzes were anonymously compiled into a mock quiz feedback document that most times exceeded 30 pages. These documents were uploaded to the MyElearning course page and made available to all students. These documents had the following for each question:

- Quiz Questions
- Student Response/Answers
- Student Grade
- Lecturer Comments/Feedback

### j. Mixing of Learning Resources

Students were required to take advantage of the use of all these learning resources (a) to (i) which were made available by the course lecturer. It was expected that the combined use of these resources will benefit students more than just a simple movement of class to online lectures using Zoom or Blackboard Collaborate. It is expected that the abundance of resources would increase the students’ chances of obtaining high marks in the course.

## Assessment of Traditional Teaching Methodology and New Teaching Methodology Prepared for COVID-19 School Restrictions

At the time in which COVID-19 school restrictions were enforced, only the midterm examination for the introductory digital electronics course was administered. The quizzes and final exam were still to be administered. Because of the COVID-19 restrictions and the uncertainty of resumption of classroom-based teaching at the university, a decision was made by the course lecturer to administer these quizzes and exams online using quizzes on the MyElearning course page. Because the midterm exam was unaffected by the COVID-19 restrictions, it was not necessary to include the results of the midterm in this article because the article focusses on those aspects affected by the COVID-19 teaching restrictions. Just for the purpose of completeness, it is to be noted that the midterm exam covered the first two learning outcomes based on Number Systems *(LO #1)* and Boolean algebra *(LO #2)*.

The first assessment affected by the COVID-19 teaching restriction was Quiz #1 which was based on Minimization with Karnaugh maps *(LO #3) and* Combinational Logic Circuits *(LO #4)* and which is normally administered as a 60-minute open-book multiple-choice quiz containing 20 questions. The multiple-choice questions were not simple as they normally required students to utilize the combinational logic procedure to arrive at the correct answers. Students cannot simply guess the correct answer. To ensure that this quiz could have been accommodated after COVID-19 teaching restrictions, the course lecturer moved the quiz from the classroom to the MyElearning course page.

**Figure 4.**
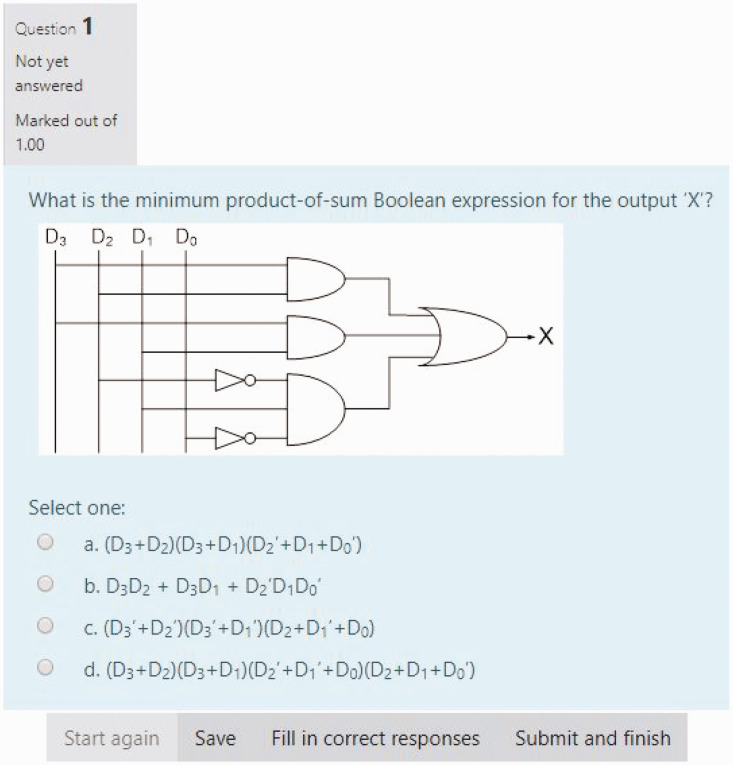
Multiple-Choice MyElearning-Based Question for Quiz #1 (Combinational Logic Circuits).

A total of 100 different multiple-choice questions were created and placed in a question bank. On the day of the quiz, 20 of these questions were randomly selected and assigned by the system to each student. Students were given 60 minutes to attempt all 20 questions. The fact that students were assigned 20 randomly selected questions and each student had a different exam of similar difficulty minimized the possibility of collusion between students. To further minimize the possibility of collusion, the available answers for each question were shuffled. The questions in the quiz were also shuffled. To ensure that issues related to availability of internet and reliability of internet source did not affect students’ progress in the quiz the quiz was run for 24 hours, students were allowed 60 minutes to attempt the quiz. After the quiz period ended, all quiz attempts were automatically graded by the MyElearning facility, and students were issued their grades. Figure 4 shows one of the multiple-choice questions administered to students in this quiz.

The second assessment affected by the COVID-19 teaching restriction was Quiz #2 which was based on an introduction to VHDL *(LO #7)* and which is normally administered as a 60-minute structured essay-type open-book quiz administered in the classroom and containing 4 questions covering the areas:

- VHDL Entities
- VHDL Architectures
- VHDL Testbenches
- VHDL Port Mapping (Component Instantiation)

To ensure that this quiz could have been accommodated after COVID-19 teaching restrictions, the course lecturer moved the quiz from the classroom to the MyElearning course page. This quiz was conducted similar to the *Online Mock Quizzes discussed in the previous section of this article*.

Twenty different structured essay-type questions (5 for each area of study) were created and placed in a question bank. On the day of the quiz, four of these questions were randomly selected (one for each area of study) and assigned to each student. Students were given 60 minutes to attempt all four questions. The fact that students were assigned four randomly selected questions served to minimizing the possibility of collusion between students.

**Figure 5.**
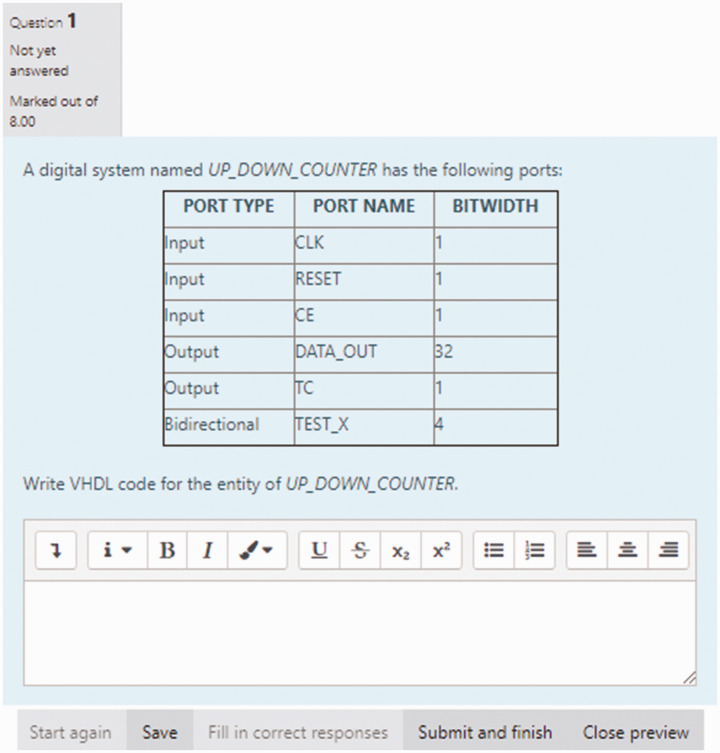
Structured Essay-Type MyElearning-Based Question for Quiz #2 (Intro to VHDL)—Entities.

**Figure 6.**
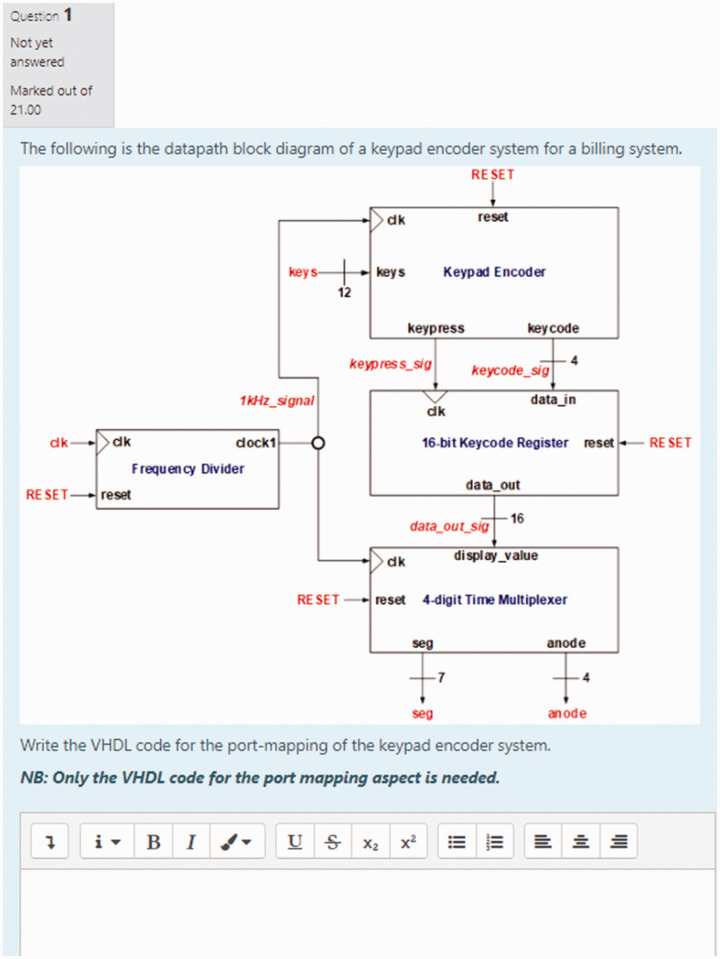
Structured Essay-Type MyElearning-Based Question for Quiz #2 (Intro to VHDL)—Component Instantiation.

To further minimize the possibility of collusion, the questions in the quiz were also shuffled. To ensure that issues related to availability of internet and reliability of internet source did not affect students’ progress in the quiz, the quiz was run for 24 hours, and students were allowed 60 minutes to attempt the quiz. Because the questions for this quiz were structured essay-type questions, the quiz attempts unfortunately could only be manually graded on the MyElearning platform. Figures 5 and 6 show some of the structured essay-type questions administered to students in this quiz.

The final exam was the last assessment affected by the COVID-19 teaching restriction and was normally administered as a 3-hour written examination with full supervision by paid invigilators and containing structured essay-type questions. The following are the topics that are normally examined:

- Minimization Using Karnaugh maps *(LO #3)*
- Combinational Logic Circuits *(LO #4)*
- Integrated Circuit Technology *(LO #5* and *LO #6)*

At the time of production of this article, the university had not as yet made a decision on how the final exam would be administered for all courses. The progress of this article could not be delayed indefinitely for the outcome of such decision so the lecturer decided to host a mock final exam of similar difficulty of the traditional final exams for the course, and it was mandatory all students attempt the quiz as a means of preparing for the final exam. The mock final exam was hosted on the MyElearning course page. This quiz was conducted similar to the *Online Mock Quizzes discussed in the previous section of this article.*

The final exam normally contained four structured questions essay-type questions containing several subquestions. Because the mock final exam was to be conducted online and to maximize student readability of exam questions while at the same time minimizing the complication of marking such exam responses, the online questions had to be arranged as stand-alone essay-type questions with no subquestions. To minimize the possibility of students colluding and also most important to allow the online exam to be conducted as similar as possible to the traditional final exam, the students had to be administered the online exam at the same time. Additional guidelines applied to the administration of this examination were as follows:

- All students attempt exam same time, and this time line must be announced at least 2 weeks in advance.
- Shuffle questions to minimize possibility of collusion by students.
- Students must have NO opportunity to return to previously attempted questions, hence minimizing possibility of collusion by students.
- Fair time budget in light of online delivery, but not excessive.
- A backup exam should also be prepared in the event students could not make it to the first on because of unforeseen circumstances.

**Figure 7.**
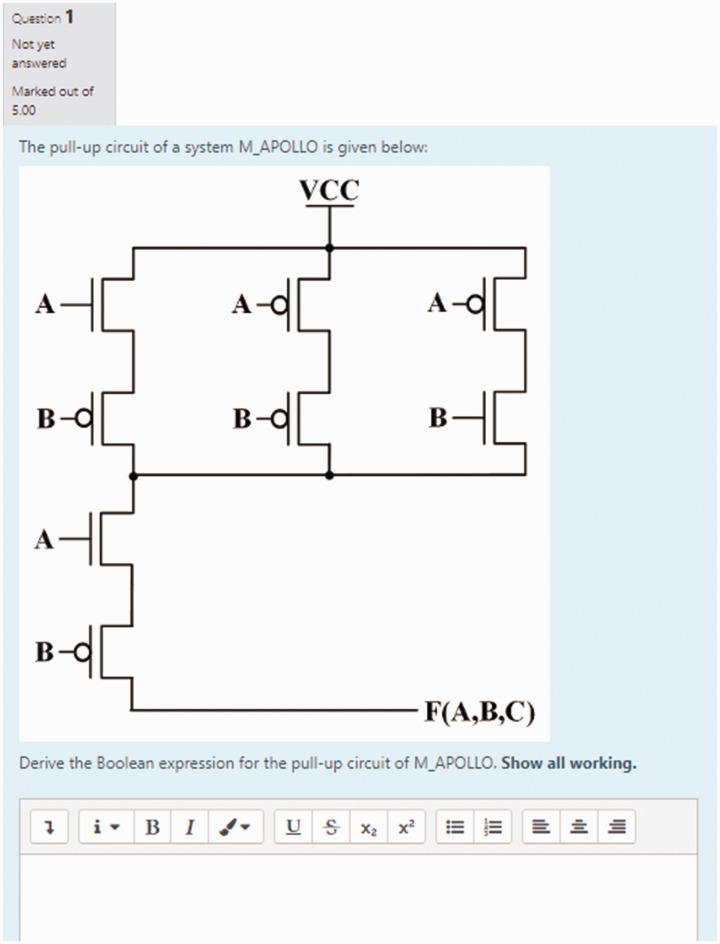
Structured Essay-Type MyElearning-Based Question for Mock Final Examination—CMOS Complements.

**Figure 8.**
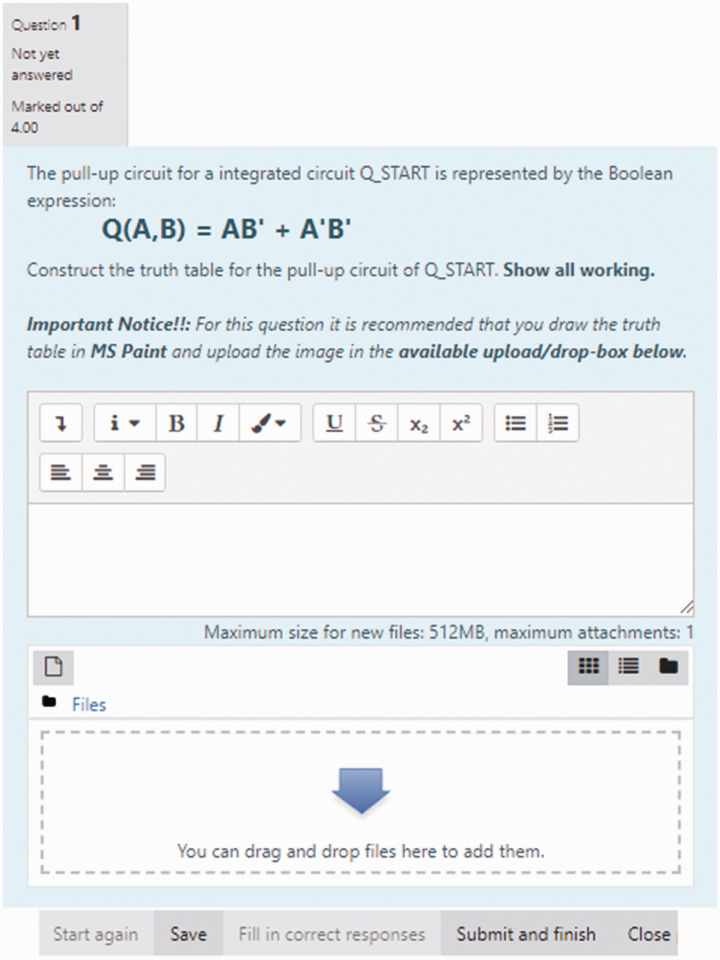
Structured Essay-Type MyElearning-Based Question for Mock Final Examination—Pull-Up/Pull-Down Circuits.

Students were required to attempt a mock final exam quiz containing seven stand-alone questions with no subquestions, and they were required to attempt this entire quiz in 2 hours, which was 1 hour less than previous years. Only one attempt was allowed. Because the questions for this quiz were essay-type questions, the quiz attempts unfortunately could only be manually graded on the MyElearning platform. Figures 7 and 8 show some of the structured essay-type questions administered to students in this mock final exam quiz.

Tables 4 [Table table5-0047239520934017]to 6 present the student performance for Quiz #1, Quiz #2, and the final exam over a 5-year period. It is important to note that the results of the mock final exam of academic year 2019/2020 were used in place of that of the eventual final exam because the university had not made a decision on how the final exams would have been conducted, and it was the author’s belief that this mock final exam gave an excellent indication of how a final exam for the course would be had it been conducted online using the strategies outlined in this article.

**Table 4.** Comparison of Student Performance in Quiz #1 (Combinational Logic Circuits) Over 5-Year Period.

Mark/8%	Student performance				
	2015/2016	2016/2017	2017/2018	2018/2019	2019/2020
7.0–8.0	26	37	32	38	42
6.0–6.9	22	22	18	20	22
5.0–6.9	21	17	13	11	9
4.0–5.9	7	1	3	4	3
0.0–3.9	2	0	1	0	0
Total	78	77	67	73	76

**Table 5. table5-0047239520934017:** Comparison of Student Performance in Quiz #2 (Intro to VHDL) Over 5-Year Period.

Mark/12%	Student performance				
	2015/2016	2016/2017	2017/2018	2018/2019	2019/2020
11.0–12.0	21	28	23	26	32
10.0–10.9	17	20	18	21	22
9.0–9.9	19	18	13	14	15
8.0–8.9	9	7	8	6	4
7.0–7.9	5	3	3	4	2
6.0–6.9	3	1	1	0	0
0.0–5.9	4	0	1	2	1
Total	78	77	67	73	76

**Table 6.** Comparison of Student Performance in Final Examination Over 5-Year Period.

Mark/60%	Student performance				
	2015/2016	2016/2017	2017/2018	2018/2019	2019/2020
50–60	24	28	11	9	31
40–49	35	29	22	13	23
30–39	12	15	19	34	16
20–29	3	3	6	11	4
0–19	4	2	9	6	2
Total	78	77	67	73	76

According to the data given in Tables 4 [Table table5-0047239520934017]to 6, it was realized that student performance in Quiz #1 and Quiz #2 were consistent with (and in some instances better than) that of their traditional counterparts of the previous academic years, despite the presence of COVID-19 teaching restriction in academic year 2019/2020 resulting in alternative teaching strategies having to be applied to prepare students for these examinations.

In the case if the final exam, Table 6 indicated that student performance in the mock final exam of year 2019/2020 was better than that of written final exams of the previous four academic years. This may have been because the mock final exam was an online exam that students were attempting without supervision, and hence students had access to reading materials to assist attempt of the exam questions. On the other end, there is no evidence to suggest that students would have performed worst if there was no possibility of accessing reading material during the online quiz.

After 1 week of submission of the original version of this article, the UWI had made a decision on how the final exam would have been administered to students; however, the exam was not to be administered until late June 2020. Traditionally, the final exam was administered as a 3-hour written examination with full supervision by paid invigilators and containing structured essay-type questions. As a result of the COVID-19 school restrictions, the university made a decision to administer the final exams as take-home final exams without supervision, where students would be allowed 48 hours to prepare handwritten or typed solutions to the questions of the exam paper, scan the solutions, and upload them on in appropriate sections of the MyElearning online platform. The student responses would then be marked by the course examiner. The author of this article however believed that the use of the mock final exam discussed earlier provided a more appropriate avenue for verifying the effectiveness of the modified teaching methodology presented in this article because it better emulated the traditional method of administering the final exam, and students were least likely to benefit from collaboration with other classmates, social media, or even internet search engines for the duration of the mock final exam.

## Course Evaluation Questionnaire for Teaching Methodologies

In each academic year 2015/2016 to 2019/2020, students were required to anonymously complete course feedback questionnaires. In the academic year 2019/2020, students were fortunate to experience both traditional and modified teaching approaches so an additional questionnaire was provided to them so they can contrast both approaches.

According to the feedback questionnaires, students in all five academic years indicated that the traditional teaching strategies benefitted them enormously in understanding of course material, and they always welcomed the opportunity to have face-to-face correspondence with the lecturer without use of devices. To have an idea of students’ opinions of the modified teaching methodology that was issued with consideration of COVID-19 school restrictions in Trinidad and Tobago, the students of academic year 2019/2020 were asked to contrast their experience under this new (modified) methodology in comparison with the traditional teaching methodology. Students indicated that the new strategies allowed students to do self-study under the guidance of the course lecturer and that this enabled them a level of convenience not allowed under the traditional teaching methodology.

Students also praised the abundance of learning resources under the new teaching methodology including visual tutors and the new course textbook prepared by the lecturer specifically for the course. Students also praised the use of mock quizzes in the new methodology that allowed them the opportunity to trial run the examination of material taught under online-exam conditions. The immediate feedback given for mistakes made were very valuable for their learning experience. Students finally indicated that the support received via the new teaching methodology eliminated their fears of failing the course because of the interruption of teaching by the COVID-19 pandemic.

At the end of the review, students rated the Digital Logic Theory for Engineers Classic Workbook, Digital Electronics Visual Tutor, Port-Mapping Tool for Digital Logic Design, and Mock Quiz Feedback Documents as the most helpful elements of the new/modified teaching methodology.

## Conclusions

This article presented effective teaching and examination strategies that can be utilized for undergraduate learning of courses during COVID-19 school restrictions. To demonstrate the use of these strategies the teaching and examination of the introductory digital electronics course of the Department of Electrical and Computer Engineering, UWI, St. Augustine campus was utilized. The article also served to demonstrate that the application of such teaching methodologies to the introductory digital electronics course avoided the student performance from degrading below what has been experienced in the past five academic years, despite the presence of COVID-19 school restrictions.

Student performance in Quiz #1, Quiz #2, and the final examination quiz were consistent with that of their traditional counterparts of the previous academic years, despite the presence of COVID-19 teaching restrictions, resulting in alternative teaching strategies having to be applied to prepare students for these examinations. Students also endorsed the use of the elements of the new teaching methodology utilized.

The success of the teaching and examination strategies of this article bring to light the possibility of moving the entire introductory digital electronics course online and facilitating a distance learning version of the course for a large market. The research serves to indicate that there is great merit in the use of online resources to support teaching of the introductory course in digital electronics at the university.

Feedback from students indicated that students may have collaborated in the learning of topics. Although the results of students under the teaching strategies outlined in this article appear to be consistent with that of previous years under the traditional techniques, there however is no evidence to indicate that students did not collaborate at all while attempting online quizzes. Although the mock final exam was conducted under stricter conditions, there is still the possibility that students could have collaborated. Future work should involve expanding the study to conduct the quizzes and mock final exam using online exam proctoring methods where the candidate is monitored during the exam, hence allowing the validation (without opportunity for doubt) of the effectiveness of the teaching strategies identified in this article in student learning of the material.

One must not discount the importance of adequate internet access for the online learning. It is important to note that the teaching and examination strategies utilized during COVID-19 teaching restrictions faced no interruptions. Both lecturer and students had adequate access to internet resources and access to the MyElearning course page. However, the methodology is not immune to interruptions. Inadequate access to internet by lecturer or students could adversely affect the use of these strategies.

The cohort of academic year 2019/2020 benefitted from both traditional and new teaching strategies during the undertaking of the introductory digital electronics course. Future works should include administering each method to independent groups of students and assessing them both via a monitored quizzes and final exams to determine which methodology resulted in better student performance. The research of this article served to indicate that the new teaching strategy does not result in the degradation of student performance below what normally is obtained. However, it will be good to know which methodology results in the better student performance and that experiment requires more control of variables.

Several lessons were learned from this experience of teaching and examination during COVID-19 teaching restrictions. The first lesson learned is the importance of presenting students with a variety of learning resources to facilitate their study of the subject area. Students learn the same subject area in a variety of ways and some methods may facilitate learning more than others, depending on the individual. In COVID-19 restrictions, students could no longer benefit from in-class lectures or face-to-face consultations with lecturers, so switching to synchronous methods of lecturing was not guaranteed to offer an effective alternative. As such, providing an abundance of learning resources and allowing students freedom of selecting the ones that best facilitate their learning was a viable decision.

The second lesson learned from this experience is that students appreciate the availability of visual tutors for their learning of the course material. Students’ use of all learning resources were tracked via the feedback questionnaire and according to this the most preferred learning resources were the Digital Electronics Visual Tutor and the Port-Mapping Tool for Digital Logic Design. The popular reason given for this preference was because of the ability to interact with these resources and obtain feedback for queries made. Lecturers from all disciplines should make an effort to incorporate visual tutors in the teaching strategy because students appear to rely on these resources outside of the classroom.

The third lesson learned is the importance of consultations with the course lecturer to student education. Whether or not there is a pandemic, students need the opportunity to approach the course lecturer for consultations, and according to George (2020), email-based consultations are the most utilized type of consultation when compared with tutorial (in class) and office-based consultation types. What was recognized during this study is students mainly used email-based consultations for clarification on matters after other avenues had been exhausted. If students found the answer using other resources, they were least likely to use email consultations, but for students who could not succeed with other resources, they eventually requested assistance via email.

The fourth lesson learned was the importance of a concise and reliable workbook for the course delivered. A workbook that concisely presents students with the most appropriate approach to attempting questions is recommended for students while outside of the classroom. Some students indicated that the textbooks utilized for other courses many times presented ambiguous explanations to questions; however, the workbooks such as George (2019) left little room for confusion. Based on this study, it can be recommended that even if a complete textbook exist for a discipline, the lecturer should still make an effort to prepare a concise workbook that serves to expand on the methodology to attempting questions that may be asked at the examination level. These books should be very clear on linking the theoretical and practical material with the decisions taken in progressing with the questions to be attempted.

The fifth lesson learned was the benefits of mock exams to both lecturers and students. Mocks exams utilized in this study served to both promote student learning of the topics being examined as well as get students acclimatized with the attempting of exams on the online platform. Mock exams give lecturers the opportunity to discover the drawbacks of examining students online. Some drawbacks realized were the difficulties of marking large volumes of student responses online compared with on paper. As such, lecturers must be more innovative in administering exams online to maximize the convenience in the marking of student response.

If this introductory digital electronics course was to be taught again online for an entire semester, the first addition would be the upgrading of the existing lecture notes with audio so that students can be guided through the notes with the voice of the lecturer. This will replace the use of online YouTube videos as presented in this study, hence eliminating the reliance on materials not developed by the lecturer. The second addition to the course must be the administering of the final exam online similar to how the mock final exam of this study was conducted.

It is unknown at this time how the COVID-19 pandemic will eventually impact college education; however, the teaching and examination strategies of this study are feasible and can be adopted for the lecturing of all disciplines even if not in a pandemic.
